# Supplementary material for: Krisp: A Python package to aid in the design of CRISPR and amplification-based diagnostic assays from whole genome sequencing data
Source: PLoS Comput Biol. 2024 May 20;20(5):e1012139. doi: 10.1371/journal.pcbi.1012139 (PMC11142669; doi:10.1371/journal.pcbi.1012139)
Supplement: S1 Text — (DOCX) [file pcbi.1012139.s001.docx]

**Supplementary Materials**

**Krisp: A Python package to aid in the design of CRISPR and amplification-based diagnostic assays from whole genome sequencing data**

Zachary S. L. Foster^1^, Andrew S. Tupper^1^, Caroline M. Press^1^, and Niklaus J. Grünwald^1^

^1^ Horticultural Crops Research Laboratory, USDA Agricultural Research Service, Corvallis, OR, 97330

**Supplemental Materials and Methods**

**Expected performance**

We estimated the theoretical expected performance of krisp_fasta. The first computationally intensive step is the extraction of candidate regions as k-mers from genome files and sorting by the conserved primer regions. The extraction of k-mers takes *O(S*A*N)* time, where *S* is the number of samples, *A* is the amplicon length, and *N* is the average number of bases in a sample’s sequence. The subsequent sorting step takes *O(S*A*N*log(N))* time because each genome file is expected to contain ‘N’ potential amplicons, which require *O(A*N*log(N))* comparison and sorting operations. Since sorting is computationally slower, the extraction and sorting of k-mers has an expected runtime complexity of *O(S*A*N*log(N)*). This means that the compute time is expected to scale linearly with the number of genome files and the amplicon length, and log-linear with mean sequence size. The other computationally intensive step is calculating the intersection of the k-mers between groups of samples. Since these k-mers are already sorted, finding conserved primer pairs can be done with a single traversal through the k-mer files. The time complexity of this step is roughly *O(S*A*N)*, as we iterate through roughly *N* amplicons and compare *S* potential amplicons across *A* different sites. This means that compute time for this step is expected to scale linearly with the number of samples, amplicon length, and mean sequence length.

We also estimated the theoretical expected performance of krisp_vcf. Since krisp_vcf reads all variants only once, processing time scales linearly with number of variants. It also should scale linearly with the number of groups being distinguished (G) since each group gets its own set of processing queues and quality filters. Finally, it should also scale linearly with the number of samples in the VCF file (S). This results in a predicted time complexity of O(V*G*S).

When multiple cores are available for computation, the real-world runtime can be significantly reduced since many of these steps are independent, or nearly independent. For example, extracting and sorting k-mers from the input files are completely independent tasks and are executed as such by krisp_fasta. Similarly, taking the intersection of k-mers is done in parallel by repeatedly taking the intersection of pairs of sample files, thereby building an inverted binary tree where the root node (i.e., the final file produced) is the intersection of all samples. Each intersection job is executed in parallel as a separate task, and each task is further written in parallel to utilize multiple cores. Krisp_vcf also takes advantage of parallel processing by splitting the input VCF file into chunks based on reference sequence coordinates, allowing for each chunk to be processed in parallel.


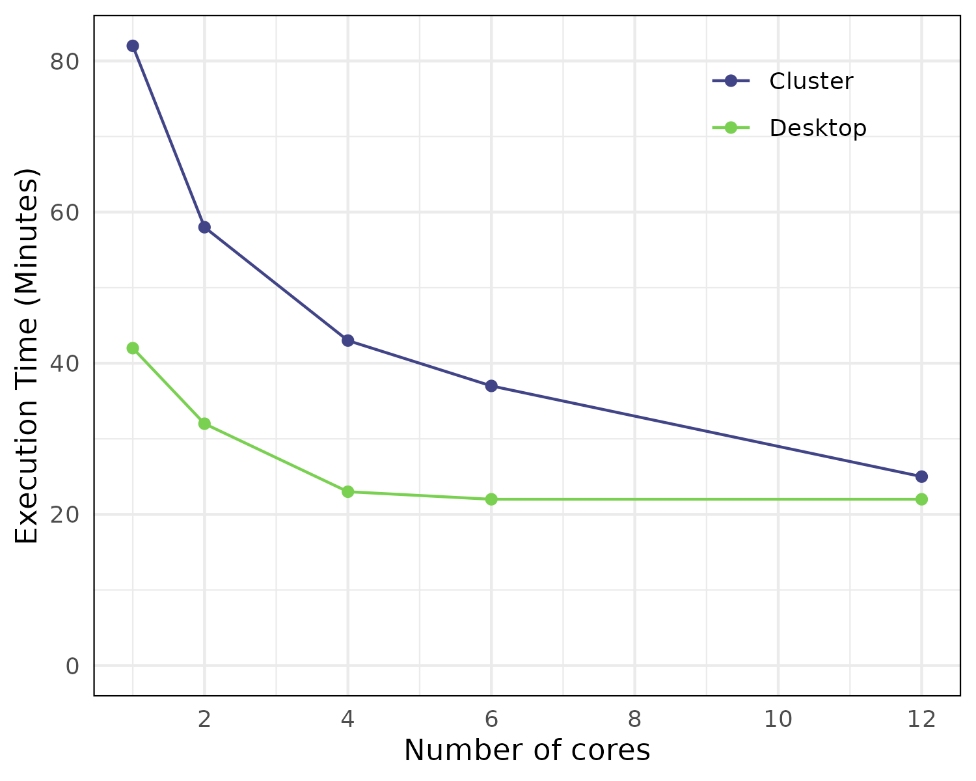


**Fig A.** Execution time of krisp_fasta on a test dataset of 12 yeast genomes with varying numbers of processors used. See Table 1 for more information on the data used. Desktop timing tests were performed on a 6-core Intel i7-8700 CPU with a clock rate of 3.20GHz and 32GB of RAM. Cluster timing tests were performed on a 64-core AMD EPYC 7992 CPU with a clock rate of 2GHz and 4GB of RAM.


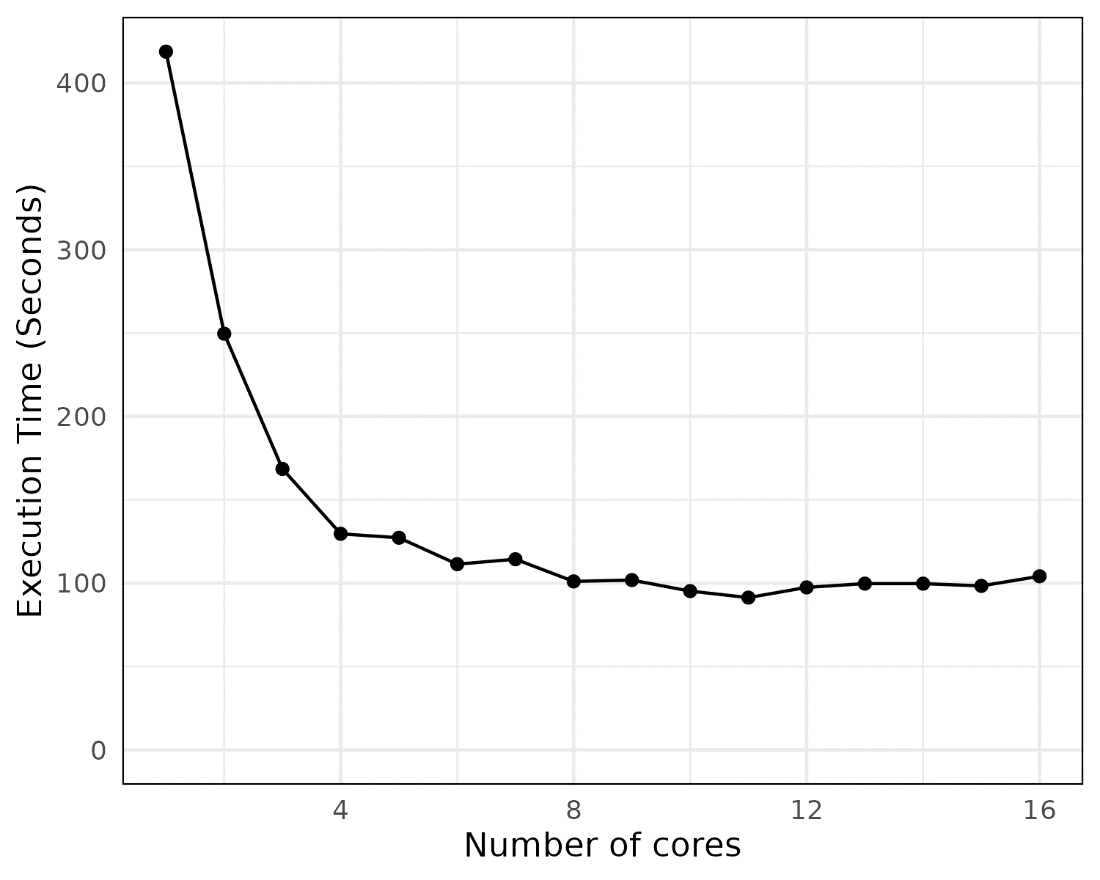


**Fig B.** The effect of the number of cores used on the execution time of krisp_vcf. The test dataset used consisted of 10 samples with 522,965 variants distinguishing 2 groups. These tests were done on a laptop computer with an Intel Core™ i7-10875H CPU @ 2.30GHz × 16 processor.
