# Supplementary material for: Krisp: A Python package to aid in the design of CRISPR and amplification-based diagnostic assays from whole genome sequencing data
Source: PLoS Comput Biol. 2024 May 20;20(5):e1012139. doi: 10.1371/journal.pcbi.1012139 (PMC11142669; doi:10.1371/journal.pcbi.1012139)
Supplement: Attachment — Submitted filename: Response to Editors and Reviewers.docx [file pcbi.1012139.s002.docx]

Dear Editors and Reviewers,

Thank you very much for the rigorous reviews of our work. We are herewith resubmitting the revised manuscript for peer review. We have addressed all comments (*in italics*) as specified below in detail (in regular font).

Sincerely,

Nik Grunwald and coauthors

*Editor:*

*As with all papers reviewed by the journal, your manuscript was reviewed by members of the editorial board and by several independent reviewers. In light of the reviews (below this email), we would like to invite the resubmission of a significantly revised version that takes into account the reviewers' comments. Particularly important is the request to expand on the description of the field and to evaluate the performance of the method in light of existing approaches.*

We agree that the manuscript would benefit from comparison with existing tools used to aid in the design of CRISPR-dx assays. There are many tools to design CRISPR guide RNAs for genome editing but few tools for designing CRISPR-based diagnostics. The few tools we found in our literature review functioned much differently than krisp, generally focusing more on the details of designing guide RNAs for particular Cas enzymes or genomes and less on large scale data analysis. Krisp was designed primarily to be fast, scalable, and generalizable to many systems. We have added to the manuscript a description of existing approaches and how they compare to krisp.

*Reviewer's Responses to Questions*

*Reviewer #1: The paper by Foster, et al. et al. describes a Python library, Krisp, that allows detection of target regions and design of primers for CRISPR applications that are able to discriminate between different strains (e.g.). The authors describe the functioning of the package, and how to use it. They cover benchmarking of the speed/CPU usage and provide a basic example of application to sequences. The paper is written well but I have the following comments for improvement.*

1. *Though the tool looks useful and the paper is well written, it is very light on any biological demonstration or interpretation. It is useful to show the P. ramorum data – and that’s great, but this doesn’t really communicate the biological impact of the approach (what would the alternative have been, e.g., in terms of primer design). The examples given are fine to given an idea of how the tool functions – but it is difficult to assess how useful the tool will be, or what kind of biological insights it might provide.*

This tool can be used for any organism (plant animal, plants, fungi, bacteria, etc.) at different taxonomic levels (e.g. genus, species, variant within species) for which assembled whole genome sequences or VCF data are available representing the genetic diversity of the samples to be distinguished. *P. ramorum* is just one proof-of-concept example. Krisp’s primary benefits over other approaches are speed, scale, and automation. While it is possible to manually identify diagnostic regions from whole genome sequences alignments, VCFs, or BAM pileups using programs with graphical user interfaces, this usually requires a priori knowledge of which genes might be useful in this regard to be done within a practical amount of time and effort. Krisp does not require the user to know which part of the genome to look at because its efficiency allows the entire genome to be searched. Additionally, krisp does not require multiple sequences alignments, which are often unreliable and difficult to produce when there are large scale genomic rearrangements. Krisp’s potential to provide biological insights relative to other methods is due to the scale of analyses enabled by its efficient use of computational resources. Krisp_vcf in particular can analyze 1000 eukaryotic genomes worth of VCF data in a few hours on a standard laptop computer. In regard to the potential for biological insights, this tool is primarily used to create other tools, namely diagnostic assays, and the use of these assays are what would yield biological insights. Krisp makes it easier to make such diagnostic assays, which will increase the number of diagnostic assays produced, which will increase the likelihood of gaining biological insights from the use of those assays. We have added more description of these benefits to the manuscript.

*2. There is a lot of benchmarking on the computational performance of the tool that is probably not necessary in the main text. I’ve included some suggestions below.*

Computational performance is the primary strength of the tool, in the sense that it allows for analyses at a scale that might have not been practical otherwise. However, we are happy to move some of this description to the supplements and have done so.

*3. Figures 2 and 3 are of limited value given that Figure 2 is only comparing the algorithm’s performance against itself, and Figure 3 can be easily summarized as text (not sure the figure adds much really).*

These figures demonstrate that krisp effectively uses multiple cores. This is relevant because not all programs that are technically “multi-threaded” actually benefit much from the use of additional cores. However, we agree that they can be summarized in the text effectively so we have moved them to the supplements.

*4. Figure 4 (bottom panel) is a bit confusing. I believe that the plot is supposed to be showing the difference between control (dashed lines) and experiment (solid lines) – but the plot looks like there are a lot more solid lines (maybe the clonal lineages) than dashed lines. I think this would be better conveyed as a bar graph (e.g.) showing the differences of all the strains at 150 minutes. The time doesn’t seem to add a lot.*

This type of plot showing fluorescence vs time is typical in papers describing the type of assays that krisp is designed to help create. The reason that there appears to be fewer dashed lines is that they all overlap at around 0. However, we agree this could be confusing. We have thus added a boxplot of fluorescence levels at time 150 minutes to try to make this clearer.

*5. Line 334, “Expected Performance” – this section does not need to be in the main text, and I feel could be moved to supplement. The expected performance is somewhat interesting, but only in light of the realized performance (in the next section).*

We have moved this section to the supplement.

*Minor comments.
6. Line 22, SHERLOCK has not been defined – so it’s an odd thing to talk about in the Abstract.*

Good point. We replaced SHERLOCK with CRISPR diagnostic assay.

*7. Line 140, WGS should be defined here.*

It was already defined above line 53, so we left it as is.

*8. Python should be capitalized throughout.*

Done.

*9. Line 258, RAM should be capitalized*

Done.

*10. Line 267, ‘P. ramorum’ has not been spelled out previously.*

Done

*11. Line 372, “On both computing systems” – both of which systems?*

Reworded as in figure 2 to desktop and cluster.

*12. Line 372, “twice as fast when 6 cores were used.” – twice as fast as what?*

Twice as fast compared to using a single core. We have clarified this in the text.

*13. Line 374, “hardware limits being reached.” – while true, not very helpful. My guess would be IO limitations? Limitations on threading relative to the algorithm?*

The laptop used had a powerful CPU for its size. We tracked the temperature of the processor and it reached maximum temperatures quickly when many threads were used. The CPU is automatically throttled at such high temperatures, causing each thread to run slower than they would if running alone. The program still runs much faster multi-threaded than it would single threaded, but it’s not a linear increase with the number of processors as would be expected if CPU throttling was not considered. The primary IO operations are only done once regardless of the number of threads, so their impacts should not scale with number of threads, but we have not tested this, so it is possible. For krisp_fasta, some of the slowdown is indeed due to some steps not being able to fully take advantage of multiple threads and we don’t know what proportion of the slowdown is due to this, but krisp_vcf should be able to take full advantage of many cores with little overhead. Since we do not know for sure why there is not a linear relationship between run time and the number of cores, we have removed speculation about this from the manuscript.

*14. Line 376, “processing the test dataset” – what was the test dataset?*

This was the yeast dataset of 12 genomes. This has been explained in the text.

*15. Line 393, “likely due to limitations of the laptop cooling system” – this seems… odd. There seem to be more likely explanations. I’m not sure any explanation without evidence is needed here.*

This reflects a downside of using laptops used for CPU-intensive tasks. Desktops have more airflow, larger fans, and larger heat sinks, which prevents the CPU from being throttled to avoid damaging temperatures. The same CPU in a laptop will run slower than if it was in a desktop due to throttling. However, we agree that this speculation is not useful and it has been removed.

*Software
1. Install from PyPi worked well*

Thanks!

*2. The Github page Requirements indicates that Python 3.6 or better is needed but it seems that the required version is Python 3.9 or better.*

Currently Python version 3.9 is required. We corrected the Github repository readme. Thanks for pointing this out.

*3. I was not able to get krispr_fasta to function with my install (latest install of Python 3.12, but other older versions still on my machine) as it seemed to be calling my Python 3.8 (which doesn’t work – see point 2, above). Though I’m sure the fix is probably relatively simple I was unable to figure it out to provide any further review of the software for the paper in a timely manner (and I’m sure others may experience similar issues).*

We have identified the issue was due to incorrectly configured Python version requirements that resulted in krisp_fasta not working on some (supported) Python versions. This problem has been fixed and krisp_fasta should work now on any supported Python version.

*Reviewer #2: The authors have developed a Python package, Krisp, capable of detecting variations common among sequences of interest. The tool provides a range of additional features, making it versatile for multiple applications and readily available for installation via Pip. The manuscript and GitHub repository are descriptive, with detailed information about the tool. There are a few comments to improve the manuscript and usability of the tool:

1) There are several existing computational tools to aid CRISPR experiments. In the introduction, The authors need to summarize the current state of computational tools in the field and the importance of Krisp, a new computational tool. How does Krisp stand out or complement them?* 

There are many tools to design CRISPR guide RNAs for genome editing but few tools for designing CRISPR-based diagnostics. Tools for genome editing typically do not incorporate primer design, do not consider non-target sequences, or only work on model organisms. The tools we found in our literature review for CRISPR-based diagnostics functioned much differently than krisp, generally focusing more on the details of designing guide RNAs for particular Cas enzymes or genomes of model organisms and less on large scale data analysis of generic data. Krisp was designed primarily to be fast, scalable, and generalizable to many systems. We have added to the manuscript a description of existing approaches and how they compare to krisp.

*2) Currently, Krisp is restricted to searching for a fixed variation length among a specified length of conserved regions. However, one could improve Krisp's versatility in diverse applications and biological data by facilitating a search of a range of lengths (e.g., 3-5 bp ).* 

If we understand this question correctly, we believe this is referring to the `--diagnostic` input of krisp_fasta and `--crrna_len` input of krisp_vcf. Indeed, this is a fixed length, but within this length there can be different numbers of diagnostic SNPs, so specifying a length of 5 for example would also find regions with 1-5 diagnostic SNPs. Krisp_vcf has the `--min_bases` option to specify the minimum number of diagnostic bases. We believe these two settings combined address the reviewer’s concern. However, we currently do not have code to specify a lower bound for the number of SNPs for krisp_fasta, but we plan to implement it in a future release.

*3) What are the drawbacks/limitations of the tool?*

Currently, krisp does not have settings or filters for optimizing crRNAs for specific Cas proteins, such as a filter for the presence of a PAM (protospacer adjacent motif). Krisp_vcf does not check for non-target amplification throughout the entire genome, only the regions mapping to the same part of the reference genome, so it may return false positives when crRNAs are designed in repetitive regions. We plan to address both of these shortcomings in the future, but they will require significant development to implement efficiently. Another limitation is that the use of Primer3 prevents it from running natively on Windows, although it can be run indirectly on windows computers with Windows Subsystem for Linux. We have added a section describing these potential drawbacks.

*Minor:
3) Can Krisp capture common variants among a set of distantly related species with deletions and insertions? One or two examples could help the readers.*

Due to krisp_fasta’s reliance on k-mers, deletions and insertions in the primer or crRNA sites could result in false negatives. However, krisp_vcf can handle deletions and insertions as long as the species are similar enough to align reads to the same reference. The ability to analyze distantly related species with krisp_vcf depends on how the input VCF was created and the capabilities of the read mapper used. We have added descriptions of these concerns in the manuscript.

*4) How does the performance scale with the number of genomes or the sequence set size? The figures on scaling to the number of cores are of little use.*

This information is plotted in the supplementary figures 1 and 2 and described in the section “Observed performance”. We have moved the figures on speed vs the number of cores to the supplements and moved the figures showing how input data size impacts performance to the main manuscript.

*5) Is the pip version of your tool the same as the current GitHub version?* 

The version is 3.9 or higher. The Github repository had a lower version that is now corrected.

*6) What are the required columns in Krisp's VCF file? Do you know if the tool works with mutated VCF file formats?*

We are not sure what the reviewer means by mutated VCF files. If the reviewer means VCF files that have been processed by other tools: We have tested krisp_vcf with VCFs produces by multiple variant callers and VCF processing tools. Krisp_vcf only relies on parts of the VCF that are defined in the format specification, so it should work with all VCF files to the best of our knowledge.

*Reviewer #3: This manuscript overall does indeed describe a reasonable approach to the goal to "find differentiating diagnostic sequences/primers for sequences of interest, in a memory efficient manner". However, this is somewhat at odds with the title and parts of the abstract, since finding primers using Primer3 is not the same thing as “A python package for designing CRISPR and amplification-based diagnostic assays from whole genome data”. For example, the described work represents what is really a front end and workflow for whole-genome primer design that depends on Primer3, not a complete package, and it does not design CRISPR guide RNAs, just primers. Having said that, overall, the work on a primer design front end and workflow is appropriate and thorough. Note the contrast here with the conclusion, that they “identify genetic regions suitable for development of DNA or RNA based diagnostic assays” – this is very different from what the they say in the title and abstract.*

We agree that the title could be rephrased more accurately, however krisp_vcf does provide suggested stretches of DNA where a guide RNA would bind to, merely requiring the user to translate that section to RNA to get the suggested guide RNA. Both krisp_vcf and krisp_fasta ensure there is a region conserved in the group of interest and different in non-target organisms where a guide can be chosen, so krisp provides resources to make choosing a guide RNA for this region quite simple. Choosing an optimal guide RNA for a given locus must take into account the particular Cas enzyme used. Although krisp may support this in the future, currently krisp is generalized to all amplicon-based diagnostics, so does not try to choose optimal guide RNAs, but rather identify regions where it is very likely that a good guide RNA and primers can be designed, while suggesting specific set of guide RNA and primers that could work in that region. We have rephrased parts of the title and abstract to reflect this distinction.

*The "chunking"/parallelization routines that the authors implemented, and demonstration of memory efficiency and discussion of performance with number of cores etc, are useful and not commonly implemented in this area.*

Nice, thanks for pointing this out.  *However the reliance on Primer3, which is rather old software with a number of known issues (for example, oversensitivity to hairpin loop structures, and inaccurate Tm calculations, at least in the versions known to this reviewer) does reduce the potential impact of the work.*

Although there were some drawbacks to using Primer3 in the past, updated versions of Primer3 claim to have solved these problems. See the following quote from “Primer3—new capabilities and interfaces”, 2012:

“The most notable enhancements incorporate more accurate thermodynamic models in the primer design process, both to improve melting temperature prediction and to reduce the likelihood that primers will form hairpins or dimers. Additional enhancements include more precise control of primer placement—a change motivated partly by opportunities to use whole-genome sequences to improve primer specificity.”

Primer3 was chosen primarily due to its speed and ubiquity. Primer3 is an open source standard for primer design and is available in websites (e.g. <https://www.ncbi.nlm.nih.gov/tools/primer-blast/>) and is also widely used in commercial software such as Geneious (<https://www.geneious.com/tutorials/primer-design-prime/>). It was designed to run with much less powerful systems than are common today, making it very fast and memory efficient. Checking for possible primers is one of the most computationally expensive steps in the program. Krisp was designed to be fast and memory efficient in order to allow for very large-scale datasets, so we wanted a fast and memory efficient way to look for primers. We expect that most people using krisp to design assays will want to modify the guide RNA and primers slightly to fit the requirements of their specific application (Cas enzyme, amplification technique, etc) anyway, so for this purpose Primer3 functions more as a way to filter out the regions clearly unsuitable for primer designed rather than suggesting optimal primers. We have added a description of these tradeoffs to the manuscript.

*Fig. 4 was also refreshing in that the authors demonstrated real-world biological performance of their method, although this demo seemed to lack needed biological context. This figure demonstrates utility in the cross-species analysis presented, but the use case for cross-species primer design is unclear, and the application seems unusual and highly specific. A better rationalization could be made for this choice of proof of concept experiment.*

Fig 4 shows the application to develop one species specific assay. The process of using krisp is identical for any other assay at any taxonomic level as long as good genome sequence data are available. We are not sure that adding another assay would enrich the work since the results would be effectively the same plots with different labels. We could for example show assays at the variant level within *P. ramorum*. The *P. ramorum* assay described produces a test to distinguish a highly regulated and destructive pathogen from closely related species. We believe this is the kind of test that would be of interest to those responsible for detecting and tracking pathogens, particularly regulatory agencies, farms, and the medical community. We have added the context to the manuscript to better explain why this example is a good representation of the kind of problem krisp is designed to solve.

*All in all, the work provides a useful platform that is useful in conjunction with other tools/experiments in a larger workflow. To be suitable for publication, the title and abstract need to better reflect the work that was actually done, the performance of Primer3 needs to be discussed, and preferably the code described needs to accommodate alternative primer design software.*

We agree that the title and abstract could be rephrased more accurately, however krisp_vcf does provide suggested stretches of DNA where a guide RNA would bind to, merely requiring the user to translate that section to RNA to get the suggested guide RNA. Both krisp_vcf and krisp_fasta ensure there is a region conserved in the group of interest and different in non-target organisms where a guide can be chosen, so krisp provides resources to make choosing a guide RNA for this region quite simple. We have rephrased parts of the title and abstract to reflect this.

Recent updates to Primer3 have addressed the reviewer’s concern (Untergasser *et al*, 2012). Primer3 was chosen primarily due to its speed and ubiquity. It was designed to run with much less powerful systems than are common today, making it very fast and memory efficient. Checking for possible primers is one of the most computationally expensive steps in the program. Krisp was designed to be fast and memory efficient in order to allow for very large-scale datasets, so we wanted a fast and memory efficient way to look for primers. We expect that most people using krisp to design assays will want to modify the guide RNA and primers slightly to fit the requirements of their specific application (Cas enzyme, amplification technique, etc), so Primer3 functions more as a way to filter out the regions clearly unsuitable for primer designed rather than suggesting optimal primers. We have added a description of these tradeoffs to the manuscript.
